# Intelligent tool wear prediction based on deep learning PSD-CVT model

**DOI:** 10.1038/s41598-024-71795-4

**Published:** 2024-09-05

**Authors:** Sumei Si, Deqiang Mu, Zekai Si

**Affiliations:** 1 College of Electromechanical Engineering, Changchun University of Technology, Changchun, 130012 China; 2 Changchun University of Technology, Changchun, 130012 China; 3 Beijing Long March Tianmin High-Tech Co., LTD, Beijing, 100176 China

**Keywords:** Convolutional neural network (CNN), Deep learning, Tool wear prediction, Power spectral density (PSD), Vision transformer (ViT), Mechanical engineering, Electrical and electronic engineering

## Abstract

To ensure the reliability of machining quality, it is crucial to predict tool wear accurately. In this paper, a novel deep learning-based model is proposed, which synthesizes the advantages of power spectral density (PSD), convolutional neural networks (CNN), and vision transformer model (ViT), namely PSD-CVT. PSD maps can provide a comprehensive understanding of the spectral characteristics of the signals. It makes the spectral characteristics more obvious and makes it easy to analyze and compare different signals. CNN focuses on local feature extraction, which can capture local information such as the texture, edge, and shape of the image, while the attention mechanism in ViT can effectively capture the global structure and long-range dependencies present in the image. Two fully connected layers with a ReLU function are used to obtain the predicted tool wear values. The experimental results on the PHM 2010 dataset demonstrate that the proposed model has higher prediction accuracy than the CNN model or ViT model alone, as well as outperforms several existing methods in accurately predicting tool wear. The proposed prediction method can also be applied to predict tool wear in other machining fields.

## Introduction

Tool wear prediction holds great importance in the field of construction machinery as it significantly contributes to improving machining efficiency, optimizing product quality, and reducing maintenance costs^1^. Intelligent manufacturing and predictive maintenance have become more popular as a result of the introduction of the Industry 4.0 concept. Deep learning technology, being a key aspect of Industry 4.0, opens up new avenues for tool wear prediction, leading to a surge in research exploring its application^2^.

In the area of predicting tool wear, there are two commonly used methods: direct measurements^3^ and indirect measurements^4^. Direct measurement methods involve acquiring image information of the tool surface using advanced visualization techniques. Through high-resolution image acquisition and detailed image analysis, these methods enable direct observation and assessment of the degree and variation of tool wear. For instance, Nguyen et al.^5^ employed a digital optical microscope and a scanning electron microscope to examine tool wear at a specific cutting distance. These non-contact direct measurement methods provide researchers with valuable visual references, offering insights into microscopic features and surface topography associated with tool wear. In contrast, indirect measurement methods estimate tool wear by collecting multiple signals generated during machining^6^. These signals include forces, vibrations, and sounds produced by the tool. With sophisticated sensors and signal processing techniques, researchers can capture and analyze the tool wear characteristics embedded in these signals. By extensively analyzing and mining the spectrum, amplitude, and waveform of the signals, it is possible to determine useful features that have a close connection to the tool wear state. Due to the numerous influencing factors involved in direct measurements during actual production machining, indirect measurement techniques are frequently utilized in tool wear prediction. These indirect methods possess advantages such as non-invasiveness, real-time capability, automation, and intelligence. They provide multidimensional information for accurate wear prediction and monitoring^7^. Although direct measurement methods still have unique advantages in specific cases, data-driven indirect measurement methods are more commonly utilized and preferred in practical applications due to the production pace and real-world machining environments.

The current state of tool wear prediction relies mainly on statistics and conventional machine learning methods like regression analysis^8^, Decision Tree^9^, Random Forest^10^, Support Vector Machines (SVM)^11^, Naive Bayes^12^, Principal Component Analysis (PCA)^13^, Hidden Markov Model (HMM)^14^, and Fuzzy Inference (ANFIS)^15^, etc. Xu et al.^16^ introduced an intelligent model called the Adaptive Neuro-Fuzzy Inference System (ANFIS) coupled with an improved Particle Swarm Optimization (PSO) algorithm to estimate tool wear.

However, the prediction of tool wear is limited by conventional machine learning techniques, including their inability to fully exploit complex features, resulting in reduced prediction accuracy and stability. These methods typically rely on manually designed feature extraction, which relies on expert knowledge and experience, making it challenging to capture implicit information in the data. Furthermore, the limited feature representation of traditional methods hinders their ability to manage massive amounts of data and intricate relationships. To overcome these challenges, researchers have turned to deep learning techniques, which have become a popular research focus for tool wear prediction in recent years.

Deep learning techniques achieve outstanding results in tool wear prediction, including convolutional neural networks (CNN)^17,18^ and recurrent neural networks (RNN)^19,20^. CNNs excel at extracting features from original data through convolutional and pooling layers, enabling the capture of local patterns and features. Pooling layers reduce the feature map size while retaining important features, and fully connected layers learn correlations between different features. Ambadekar et al.^21^ performed a triple classification task on rear tool face surface texture features to determine the wear status. In addition, based on this architecture of the RNN model, the short-term memory (LSTM)^22,23^ and the gated recursive unit (GRU)^24^ are derived, which establish a temporal relationship. Zhou et al.^25^ combined wear features and operating conditions using LSTM to predict tool life. In recent years, Transformers^26–28^ have gained attention and have been applied to various engineering fields due to increased computational power. The CNN-Transformer neural network (CTNN) model, proposed by Liu et al.^29^, aims to estimate wear unsupervised by processing data in parallel and learning variance. Li et al.^30^ introduced an IE-SBiGRU model that generates long time series feature sequences from multiple signals to achieve global awareness and long-distance parallel operations for tool wear prediction.

However, each of these methods has its drawbacks in engineering applications. CNNs have limitations in capturing global features^31^, traditional RNNs struggle with the long-term dependency problem^32^, and LSTM and GRU lack parallel computation capabilities^33^. Transformers, widely used in natural language processing (NLP)^34^, excel in processing sequential data with long-range dependencies and parallel computing capabilities. Transformer variants such as GPT^35^, BERT^36^, XLNet^37^, T5^38^, and ViT^39^ have shown success in other domains. However, using large Transformer-based models in engineering problems, where data is often limited, may lead to overfitting and increased computational resource requirements.

To solve the problem of complementary combination of local and global features in the tool wear prediction process, a new deep learning model PSD-CVT is proposed in this paper, which uses multi-channel sensor signals to generate power spectral density (PSD) maps and enhances the prediction capability by extracting key features from local and global perspectives through CNN and ViT, respectively. Validation conducted on the PHM2010 dataset demonstrates the favorable performance of the proposed model.

The following are the primary contributions of this paper:

1. A PSD-VCT model for tool wear prediction is proposed, which provides an ingenious and effective approach by utilizing PSD for data transformation processing and utilizing the benefits of CNN and ViT for feature extraction.
2. Tool wear prediction is performed using the novel deep learning model, which provides a new solution in this field.
3. Results from the experiments on the PHM2010 dataset demonstrate that the model can extract not only key features from a local perspective but also global features by capturing long-distance dependencies in images through a self-attention mechanism, thus achieving high accuracy in wear prediction.

What follows is the remainder of this paper: section “Related work” gives background information on the method and related work on data processing and model building. In section “Research method”, a detailed description of the advantages of the proposed tool wear prediction method, and its model structure is provided. Section “Experiment study” presents the experimental and analytical results obtained from the PHM2010 dataset and the comparison model. Finally, Section “Conclusions and future works” summarizes the research findings and offers a reference for future research.

## Related work

### Power spectral density (PSD)

The signal is transformed from the time domain to the frequency domain through the application of the Fourier transform. By employing the Fourier transform, a signal can be broken down into a series of sinusoidal or complex exponential components of varying frequencies. The PSD is the square of the amplitude spectrum of the Fourier transform result, and the formula is shown below:

1 $$S_{XX} \left( f \right) = \left| {\mathop \smallint \limits_{ - \infty }^{\infty } x\left( t \right) \cdot e^{ - 2\pi ift} dt} \right|^{2}$$

where $S_{XX} \left( f \right)$ denotes the PSD of the signal, and $x\left( t \right)$ is the signal in the time domain.

PSD plots play a crucial role in spectral analysis as they effectively depict the energy distribution of a signal at different frequencies. These plots offer an intuitive representation of the frequency domain characteristics of the signal, aiding in the comprehension of its spectral features. In comparison to directly utilizing Fourier transform results, PSD plots emphasize the frequency components, making the spectral characteristics more apparent and facilitating the analysis and comparison of different signals. Additionally, PSD plots are often subjected to smoothing techniques to minimize the impact of noise interference and enhance the readability of the graph. In essence, PSD plots serve as a valuable tool for comprehensively understanding the spectral shape and frequency domain properties of signals. Consequently, they are widely employed in spectral analysis and signal processing endeavors.

### Convolutional neural network (CNN)

**Fig. 1:**
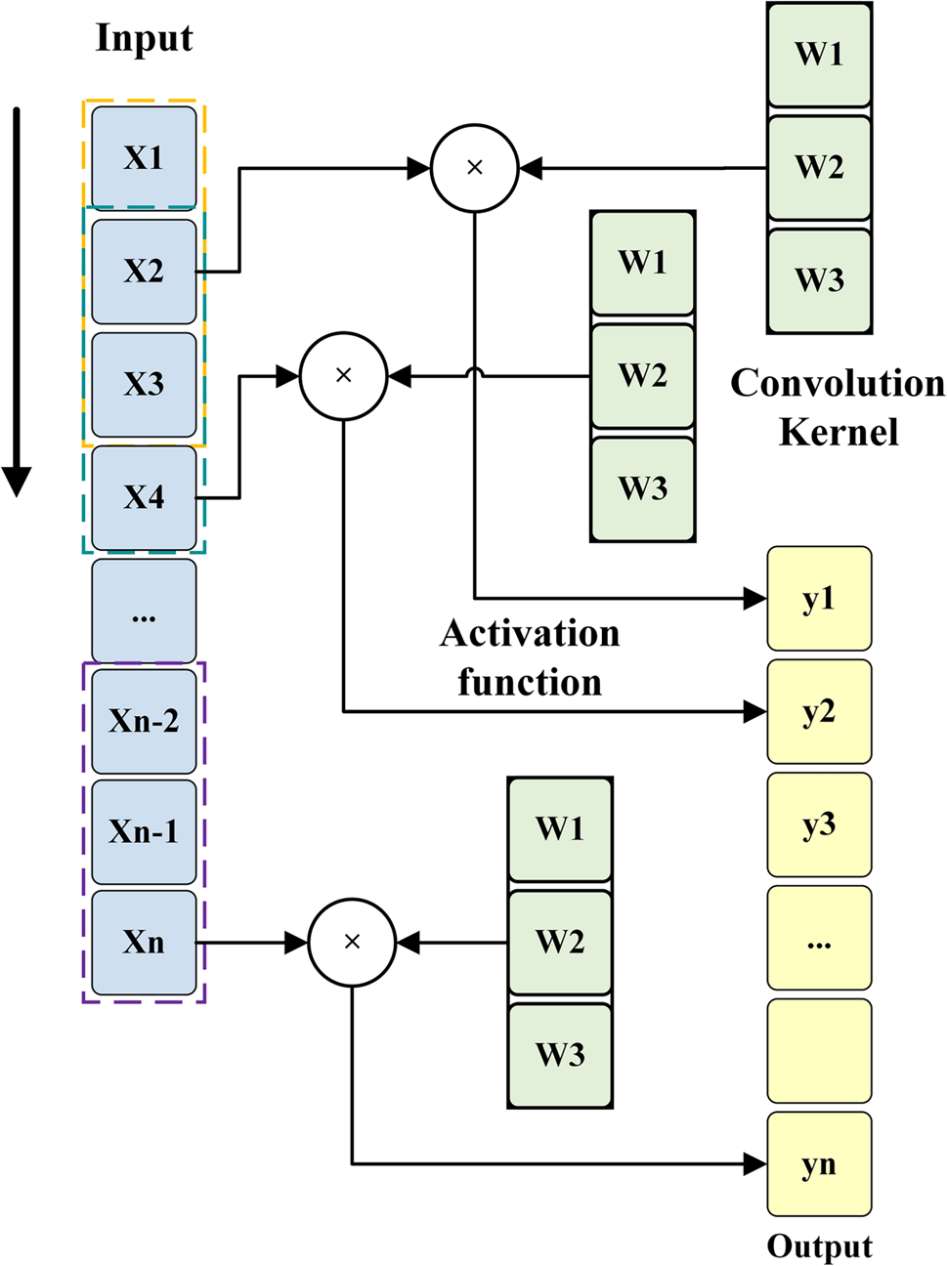
1-D convolution.

CNN is a well-known architecture in the field of neural networks, particularly effective for handling data with spatial structures. In the context of analyzing and processing signals related to tool wear processes, where multi-signals are transformed into PSD maps, CNNs prove to be a suitable choice. Features are extracted and analyzed from the input data in the CNN model through a combination of convolutional and pooling layers. In the following section, a brief overview of the CNN architecture is illustrated in Fig. 1.

Convolutional Layer: The convolutional layer is a crucial component of CNNs. It applies a learnable convolution kernel, also known as a filter, to the input image through a convolution operation. This process calculates the result of the convolution operation at each position. By convolving the kernel over the entire image, local features are extracted from the input. The convolution operation involves element-wise multiplication of local regions of the input image with the convolution kernel. The products are then summed to obtain an output value. Mathematically, this can be expressed as follows:

2 $$(I*K)_{a,b} = \mathop \sum \limits_{k,l} I_{a + k,b + l} \cdot K_{k,l}$$

where I is the input feature map, K is the convolution kernel, $(I*K)_{a,b}$ denotes the elements in the output feature map, $k,l$ are the index of the convolution kernel, and $a,b$ are the index of the output feature map.

Activation Function: An activation function is applied to the output of a convolutional layer to introduce nonlinearity. The ReLU function enhances the expressiveness of the network by performing an element-by-element nonlinear transformation of the output of the convolutional layer. It can be defined as follows:

3 $$f\left( x \right) = {\text{max}}\left( {0,x} \right)$$

where $f\left( x \right)$ denotes the result from the function of activation, and $max\left( {0,x} \right)$ denotes taking the larger value between 0 and $x$.

Pooling Layer: The output of the convolutional layer is spatially down-sampled using the pooling layer. This downsampling process reduces the number of parameters and computational complexity while extracting more robust features. One commonly used pooling operation is MaxPooling, which divides the feature map into non-overlapping regions using a $2{*}2$ pooling window. Then, the maximum value within each region is taken as the output. The MaxPooling operation can be expressed as follows:

4 $${\text{MaxPooling}}\left( x \right) = max\left( {x_{a,b} ,x_{a + 1,b} ,x_{a,b + 1} ,x_{a + 1,b + 1} } \right)$$

where $x_{a,b}$ denotes the elements within the pooling window.

### Vision transformer (ViT)

**Fig. 2:**
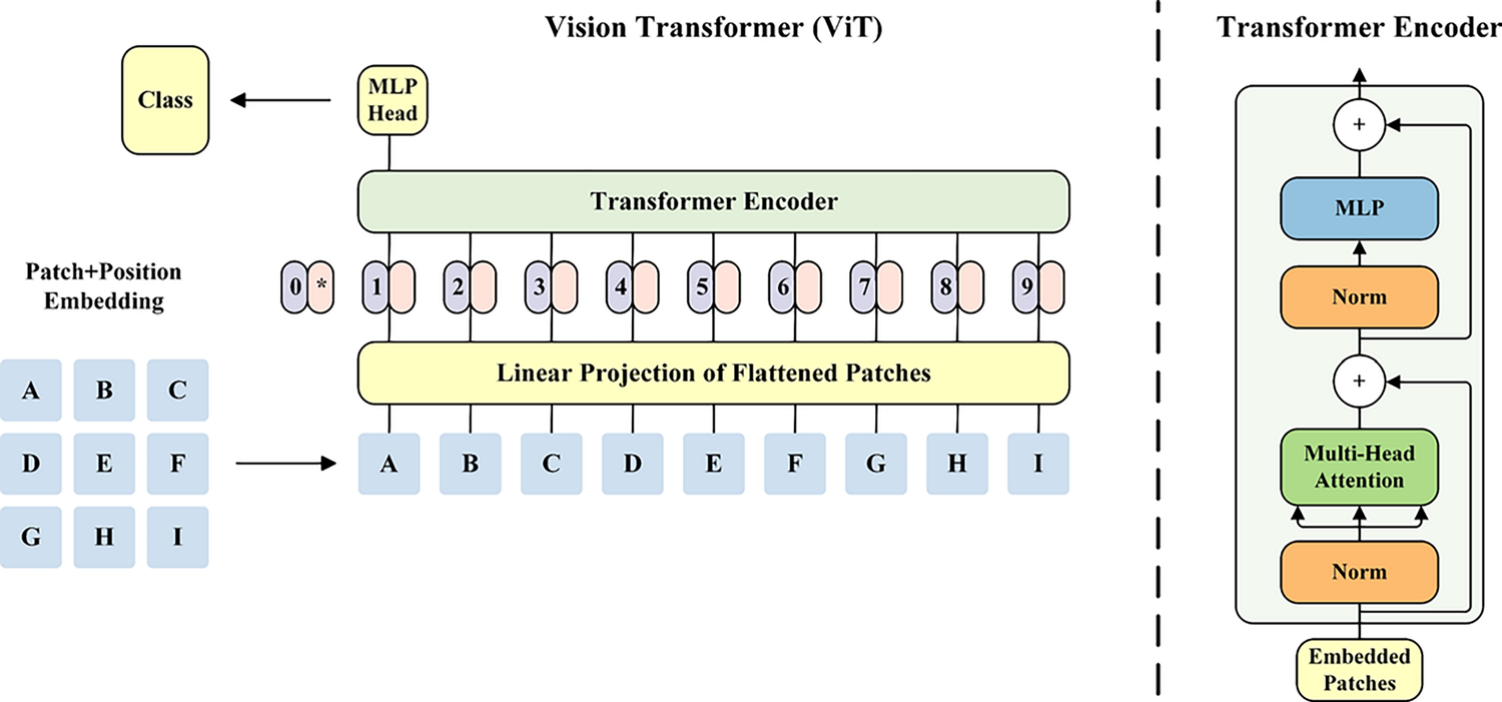
Network structure of ViT.

ViT is a computer vision model that adopts the Transformer architecture for tasks like image classification, target detection, and semantic segmentation. Its structure is depicted in Fig. 2. ViT leverages the capabilities of a Transformer and treats an image as a sequence, akin to text sequences in natural language processing.

Input representation: In ViT, the image is broken up into fixed-size image blocks that are then vectorized and embedded in a lower-dimensional feature space. Consequently, the image is represented as a sequence with a size of $N*D$, where N denotes the number of image blocks, and D represents the vector dimension of each block.

Embedding Layer: The embedding layer in ViT employs a simple linear transformation to convert the $N*D$ input image sequence into a lower-dimensional $N*d$ embedding sequence. This transformation can be mathematically expressed as follows:

5 $$Ember\left( {x_{i} } \right) = W_{e} x_{i} + b_{e}$$

where $x_{i}$ is the i-th image block in the input sequence, $Ember\left( {x_{i} } \right)$ is the corresponding embedding vector, and $W_{e}$ and $b_{e}$ are the learnable parameters.

Positional Encoding: In the ViT, positional encoding plays a crucial role in associating each embedding vector with its corresponding position in the input image. To achieve this, a commonly used approach involves generating a fixed set of positional encoding vectors through the utilization of sine and cosine functions. The attention mechanism in ViT can be represented by Eq. (6):

6 $$Attention\left( {Q,K,V} \right) = softmax\left( {\frac{{QK^{T} }}{{\sqrt {d_{k} } }}} \right)V$$

where $Q$ is a query matrix, $K$ is a key matrix, and $V$ is a value matrix.

After the self-attentive layer, the features at each location undergo a nonlinear transformation using a feedforward neural network. This neural network typically comprises two fully connected layers with an activation function, such as ReLU, and a batch normalization layer placed between these two layers.

## Research method

**Fig. 3:**
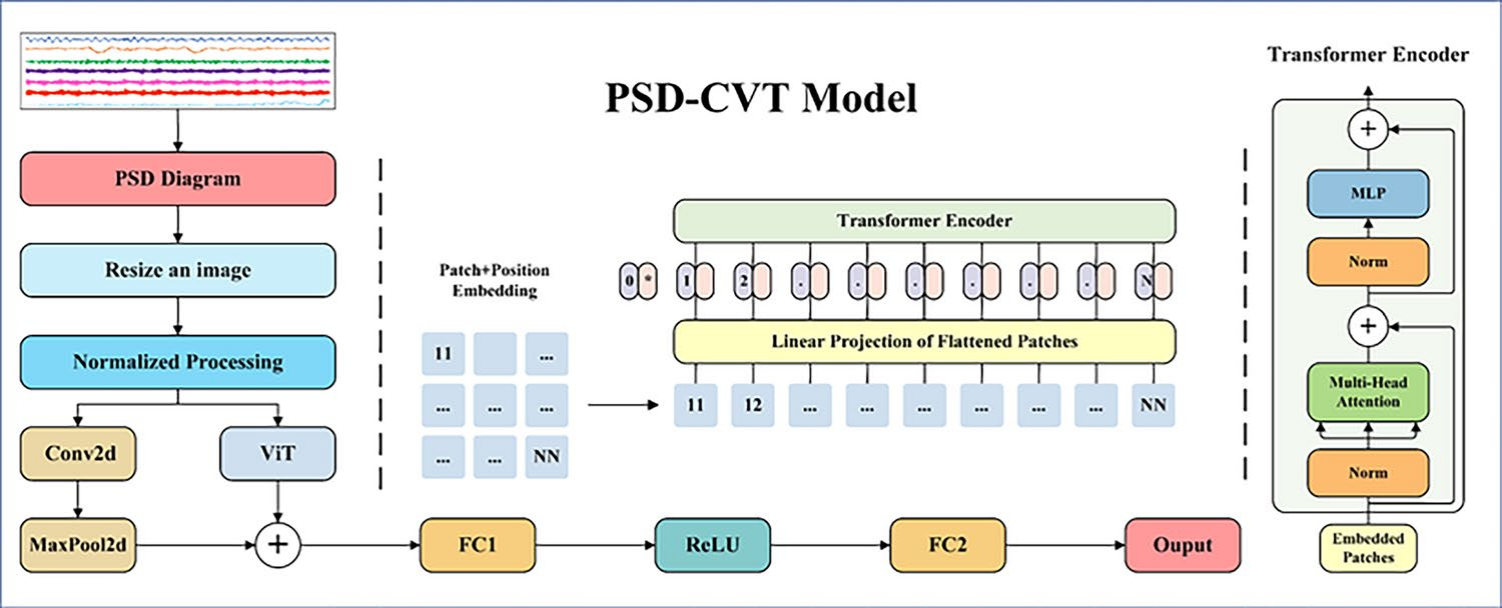
Overall structure of PSD-CVT.

This section presents the model architecture based on PSD-CVT, and the corresponding flowchart is depicted in Fig. 3. The signals captured by each sensor undergo a conversion process, transforming them into a PSD map. Subsequently, a normalization operation is applied to adjust the size of the PSD image to $224{*}224$. For further processing, the input is subsequently divided into two parts.

In the first part, a $3*3$ convolution kernel is applied to the input image to perform the convolution operation, resulting in a feature map with 32 channels. This operation aims to extract image features. The convolved feature map is subsequently downsampled using a maximum pooling layer, reducing the size by half.

The second part incorporates the ViT module. The ViT-B/16 model, which has been pre-trained for large-scale image tasks, is employed in the ViT module, giving it powerful image feature extraction and generalization capabilities. Its effectiveness has been verified on different tasks and domains through extensive training and validation on numerous datasets.

The $16{*}16$ size patches are also used because using larger image blocks as input may increase the computational and memory requirements, leading to more complex and inefficient models. To balance computing resources and performance needs, a smaller image block size is chosen. Initially, an image segmentation layer (Patch Embedding) is applied to segment the input image into a set of $16{*}16$ size image blocks. Each image block is transformed into a vector through a linear transformation to capture block-level features. To preserve positional information, sine, and cosine functions are utilized to generate position encoding. Next, the core part of the ViT model is introduced, which consists of multiple encoder layers. Each encoder layer comprises self-attention^40^ mechanism sub-layers and feedforward network sub-layers. Self-attention mechanism sub-layer adaptively computes the weight of each patch based on its relationship with other patches, facilitating the capture of global dependencies. The feedforward network sub-layer performs nonlinear transformations on each patch, ensuring a consistent mapping of the output content.

Finally, the convolved feature vectors from the previous part are then concatenated with the feature vectors from the ViT module along the first dimension, resulting in a more comprehensive feature representation. A fully connected layer (FC1) is used to process the concatenated feature vectors, resulting in an output size of 256. The output of FC1 is then passed through the ReLU activation function. Subsequently, the resulting output is fed into another fully connected layer (FC2) with an output size of 1. Finally, the output of FC2 is returned as the prediction result.

The multi-channel sensor signal is converted into a PSD map by a PSD-CVT model to capture the frequency domain characteristics. This conversion allows the model to understand the frequency distribution of the signal and thus analyze its periodicity and frequency characteristics. Secondly, the CNN model is good at extracting detailed local features, while the ViT model is fast at capturing the global relationship between pixels using a self-attention mechanism with powerful global sensing capability. By fusing these architectures, the PSD-CVT model can effectively consider both local and global features to achieve a more comprehensive analysis and processing of signal data.

## Experiment study

### Introduction to the baseline dataset

The effectiveness and high accuracy of the proposed PSD-CVT model are demonstrated through experimental model training using the PHM2010 dataset, which involved the use of a 3-flute ball-tipped carbide milling cutter on a Roders Tech RFM760 high-digit CNC machine. For the experiment, the following cutting conditions were applied: 10,400 revolutions per minute (rpm) spindle speed, 1555 mm per minute (mm/min) feed rate, 0.2 mm axial depth of cut, 0.125 mm radial width of cut, and 0.001 mm feed per journey. During the machining process, various sensors were employed to measure different signals. A three-way force gauge was used to capture force signals, an acoustic emission sensor was used to record acoustic emission data, and three accelerometers were used to monitor vibration signals. These seven signals were gathered with an NI DAQ data acquisition device at a frequency of 50 kHz. After each machining stroke, the three-edge wear was simultaneously assessed with a LEICA MZ12 microscope. Data sets C1, C4, and C6, which included the data of the entire cycles, were chosen for this experiment and utilized as the training and validation data sets. Each data set contains one “wear” file that lists wear after each cut in 10^–3^ mm and a folder with 315 individual data acquisition files (one for each cut).

### Data processing

**Fig. 4:**
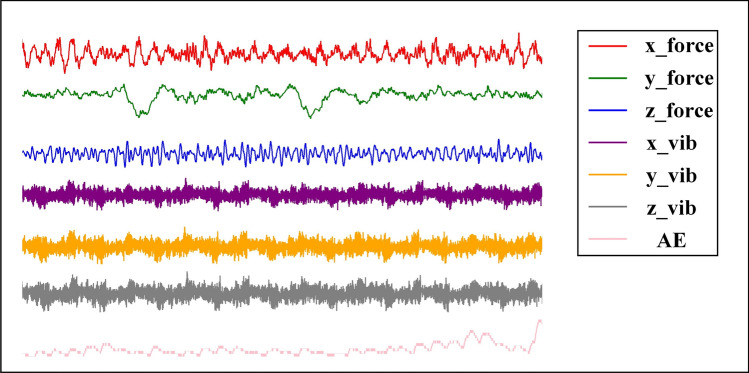
The seven signals collected.

**Fig. 5:**
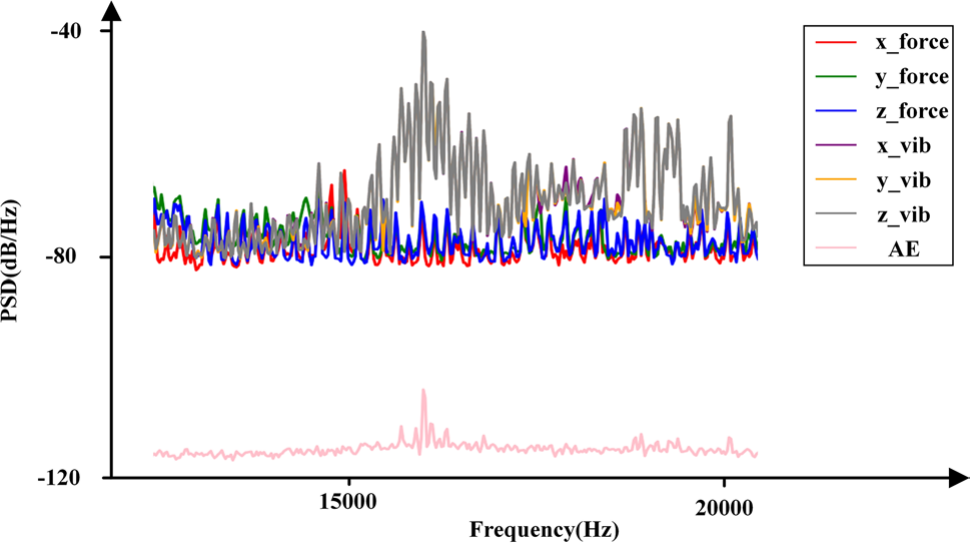
PSD part of the frequency domain detail map.

The tool generates seven signals in each stroke, as shown in Fig. 4. By calculating the square of the amplitude spectrum of the Fourier transforms result, the PSD image was obtained, which is shown in Fig. 5. Subsequently, the PSD map of each stroke was adjusted to a specified size of $224*224$ and normalized. This processed PSD map served as the input for the subsequent ViT with the CNN part of the model. In terms of the experimental setup, the wear label is selected based on the average value of the wear on the three sides. To create individual datasets for the experiments, the original data set is divided into an 80% training set, a 10% validation set, and a 10% test set. This division ensured that each dataset was appropriately split for training, evaluating, and testing the PSD-CVT model.

**Fig. 6:**
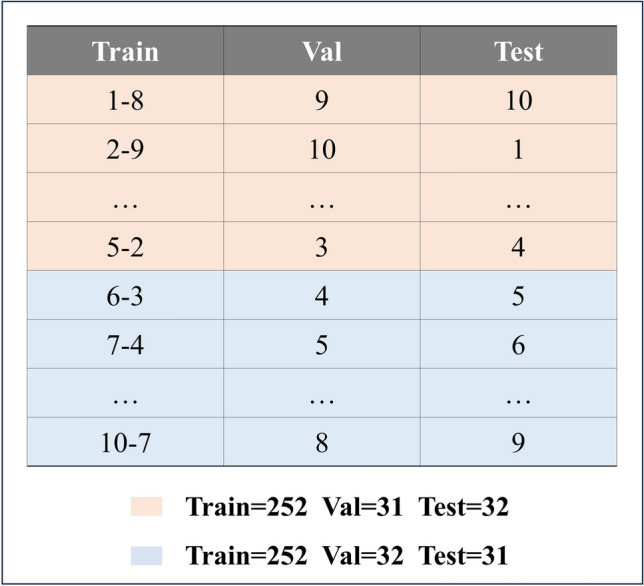
Data partitioning.

To ensure a comprehensive assessment and to fully utilize the entire dataset, cross-validation was employed. In this method, the dataset was divided into multiple subsets, and the model was trained and tested multiple times, with each subgroup serving as the test set in one of the iterations. This approach allowed for the use of all data points for both training and evaluation, thereby providing a robust estimate of the performance of the model. As shown in Fig. 6.

### Parameter and hyper-parameter settings

Table 1. provides the parameters for each layer of the model. The optimizer used for the model is Adam^41^.In the case of regression problems, the loss function usually selects mean square error (MSE). Because the smaller the value of MSE, the better the model fits. The average value of the MSE on each training batch is calculated as an evaluation metric to assess the performance of the model:

7 $$MSE = \frac{1}{n}\mathop \sum \limits_{i = 1}^{n} (y_{i} - \hat{y}_{i} )^{2}$$

where $n$ is the sample size, $y_{i}$ is the true value, and $\hat{y}_{i}$ is the predicted value.

**Table 1:** PSD-CVT model parameters.

Layers	Parameters
ViT	Transformer layers: 4 Attention Heads: 8 Hidden:256
Convolutional	Input channels: 3, Output channels: 32, Kernel size: 3 × 3, Padding: 1
Max pooling	Kernel size: 2 × 2, Stride: 2
Linear	Layer number:2, hidden dim per layer:256,1

To address the common issue of overfitting in the training of neural network models, this study employs the early stopping strategy^42^. This strategy is a crucial technique for preventing model overfitting during the training process. The basic idea of this strategy involves setting an early stopping patience value (*n*). During the training process, if there is an improvement in validation loss within the patience range, the patience value is reset to zero, and the training continues. If the validation loss does not decrease for *n* consecutive training epochs, it is considered that the model has started to overfit. At this point, the early stopping strategy is triggered to prevent further decline in the performance on the validation set. The StepLR scheduler reduces the learning rate of the optimizer by a factor (gamma) every few epochs (step size), helping the model to converge more efficiently and potentially improving its performance by making the learning rate adjustments more gradual. Table 2 presents the specific values of these hyperparameters.

**Table 2:** Hyperparameter settings.

Hyper-parameters	Learning rate	Step size	Gamma	Epoch number	Batch size	Maximum patience value
Values	0.001	10	0.5	1000	8	4

### Evaluation metrics

In previous studies, the mean absolute error (MAE) and root mean square error (RMSE) are commonly utilized as performance metrics for prediction problems. These metrics provide quantitative measures of prediction accuracy. The MAE and RMSE can be calculated using the following equations:

8 $$MAE = \frac{1}{n}\mathop \sum \limits_{i = 1}^{n} {\mid }y_{i} - \hat{y}_{i} {\mid }$$

9 $$RMSE = \sqrt {\frac{1}{n}\mathop \sum \limits_{i = 1}^{n} (y_{i} - \hat{y}_{i} )^{2} }$$

where $n$ is the sample size, $y_{i}$ is the true value, and $\hat{y}_{i}$ is the predicted value of the model.

### Result discussion and comparison

**Fig. 7:**
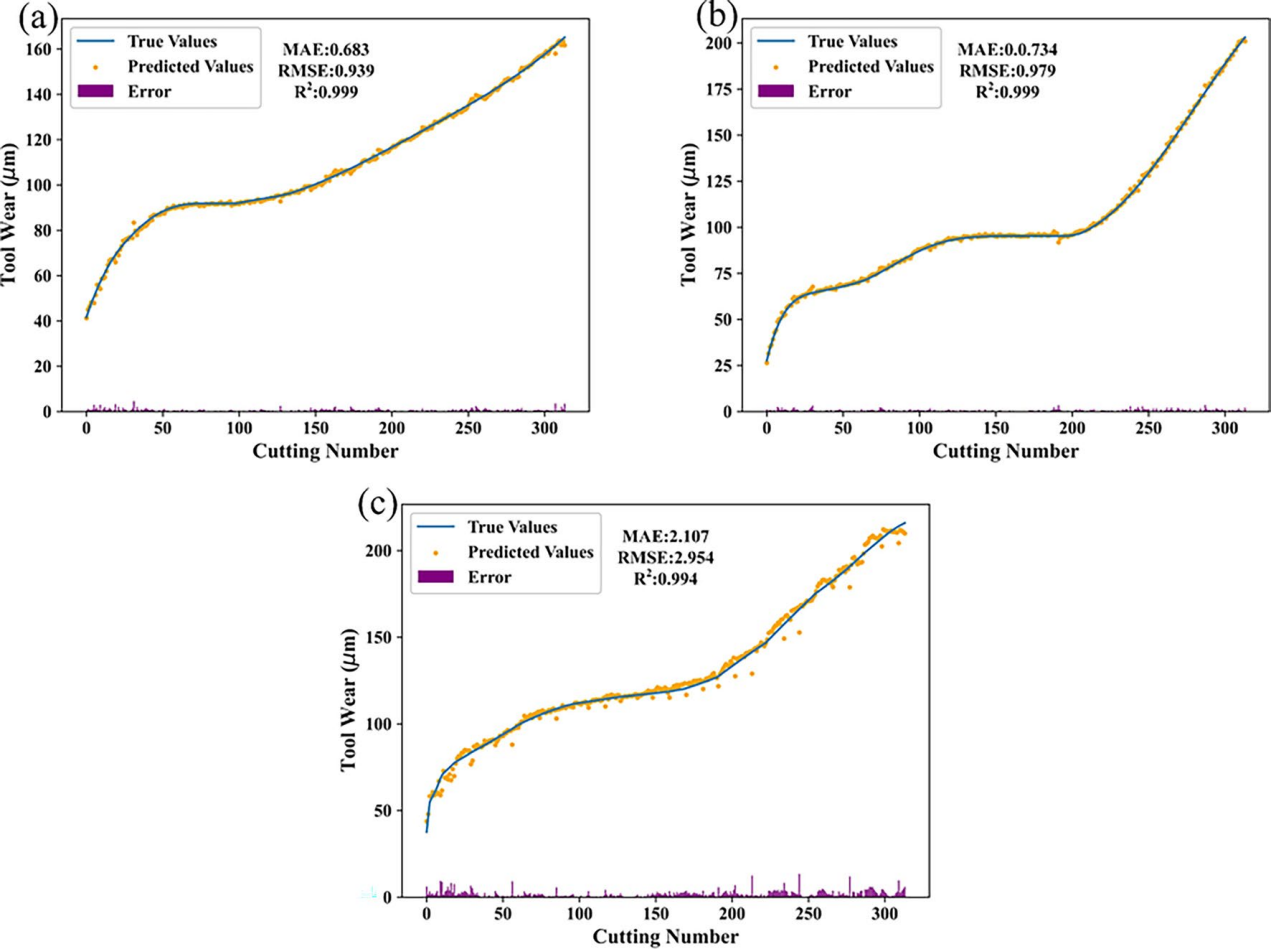
The wear prediction results of the PHM2010 testing dataset for the proposed PSD-CVT model: (**a**) predicted wear of C1 cutter, (**b**) predicted wear of C4 cutter, (**c**) predicted wear of C6 cutter.

The trained PSD-CVT model is applied to the raw data for wear prediction, resulting in accurate wear prediction results. Figure 7 illustrates the high agreement observed between the predicted wear curves generated by the model and the actual curves. This demonstrates that the PSD-CVT model is capable of effectively performing the prediction task related to tool wear. The close match between predicted and actual wear curves highlights the accuracy of the model and its ability to accurately capture and predict wear patterns.

In this paper, the PSD-CVT model is compared with the CNN model and ViT model alone, as well as some existing models. The experimental results, presented in Table 3, prove the effectiveness of fusing frequency domain features through PSD. By combining the local feature extraction capability of CNN and the global perception capability of ViT, the PSD-CVT model achieves significantly improved prediction accuracy. The CNN model excels in extracting local features and spatial information, while the attention mechanism in the ViT model enables effective modeling of global features. By leveraging both local and global features, the PSD-CVT model demonstrates enhanced accuracy in tool wear prediction tasks. This finding underscores the importance of integrating different components and operations in deep learning models. Comparisons with published papers further support the superior performance of the PSD-CVT model. The experimental results highlight the potential of leveraging PSD data pre-processing and the complementary advantages of ViT and CNN in improving tool wear prediction accuracy. This research provides valuable insights for optimizing model architectures and underscores the practical applications of deep learning models in tool wear prediction and related tasks. In conclusion, the experimental outcomes verify that the proposed PSD-CVT model is effective. And emphasize the significance of integrating different components and operations in deep learning models. This research contributes to the advancement of tool wear prediction models and opens avenues for further exploration and optimization of model architectures.

**Table 3:** Performance analysis of different models.

Model	Datasets					
	MAE			RMSE		
	C1	C4	C6	C1	C4	C6
MLP^20^	24.5	18.0	24.8	31.2	20.0	31.4
Deep LSTMs^20^	8.3	8.7	15.2	12.1	10.2	18.9
TBNN^43^	4.294	–	7.772	6.116	–	7.835
CTNN^29^	3.634	–	7.531	5.358	–	9.209
IE-SBIGRU^30^	3.694	5.189	3.398	5.056	6.884	4.527
CNN	1.234	3.988	2.467	1.600	6.113	3.384
ViT	23.36	27.71	33.94	27.54	37.67	40.12
PSD-CVT	0.683	0.734	2.107	0.939	0.979	2.954

## Conclusions and future works

A new PSD-CVT model is proposed in this paper for predicting tool wear, which combines the benefits of PSD, CNN, and ViT architectures to achieve accurate tool wear prediction. The proposed scheme aims to enhance machining efficiency, improve quality, and reduce production costs in the tool wear machining field. By converting force, acceleration, and acoustic emission signals into PSD images and utilizing the CNN and ViT encoder for feature extraction, the PSD-CVT model demonstrates superior performance compared to other researchers and individual CNN or ViT-based approaches. The experimental results of the PHM2010 dataset strongly demonstrate the effectiveness and high accuracy of the scheme in capturing the unique characteristics of tool wear and accurately predicting wear. The contributions of this research are as follows:

1. The proposal of the PSD-CVT scheme introduces a novel approach that intelligently combines PSD, ViT, and CNN techniques for tool wear prediction.
2. The feasibility of the scheme is experimentally verified, highlighting its superior performance compared to some existing methods.
3. These findings hold promising prospects for advancing machining intelligence and provide help for future research and advancements in related fields.

The future should focus on further improving the performance of the model in real production environments and exploring practical applications. Overall, the proposed method provides a reliable and innovative approach to tool wear prediction that has implications for a variety of industries and applications.

## Data Availability

The datasets used and/or analyzed during the current study available. They are come from the PHM 2010 dataset (https://phmsociety.org/phm_competion/2010-phm-society-conference-data-challenge).
